# Supplementary material for: The interactome of intact mitochondria by cross-linking mass spectrometry provides evidence for coexisting respiratory supercomplexes
Source: Mol Cell Proteomics. 2017 Dec 8;17(2):216–32. doi: 10.1074/mcp.RA117.000470 (PMC5795388; doi:10.1074/mcp.RA117.000470)
Supplement: Supplemental Data [file supp_RA117.000470_133922_0_supp_23978_fzffwf.zip › spectra_annotation/mito_spectra_annotation/473-1-11-1-10-1.pdf]

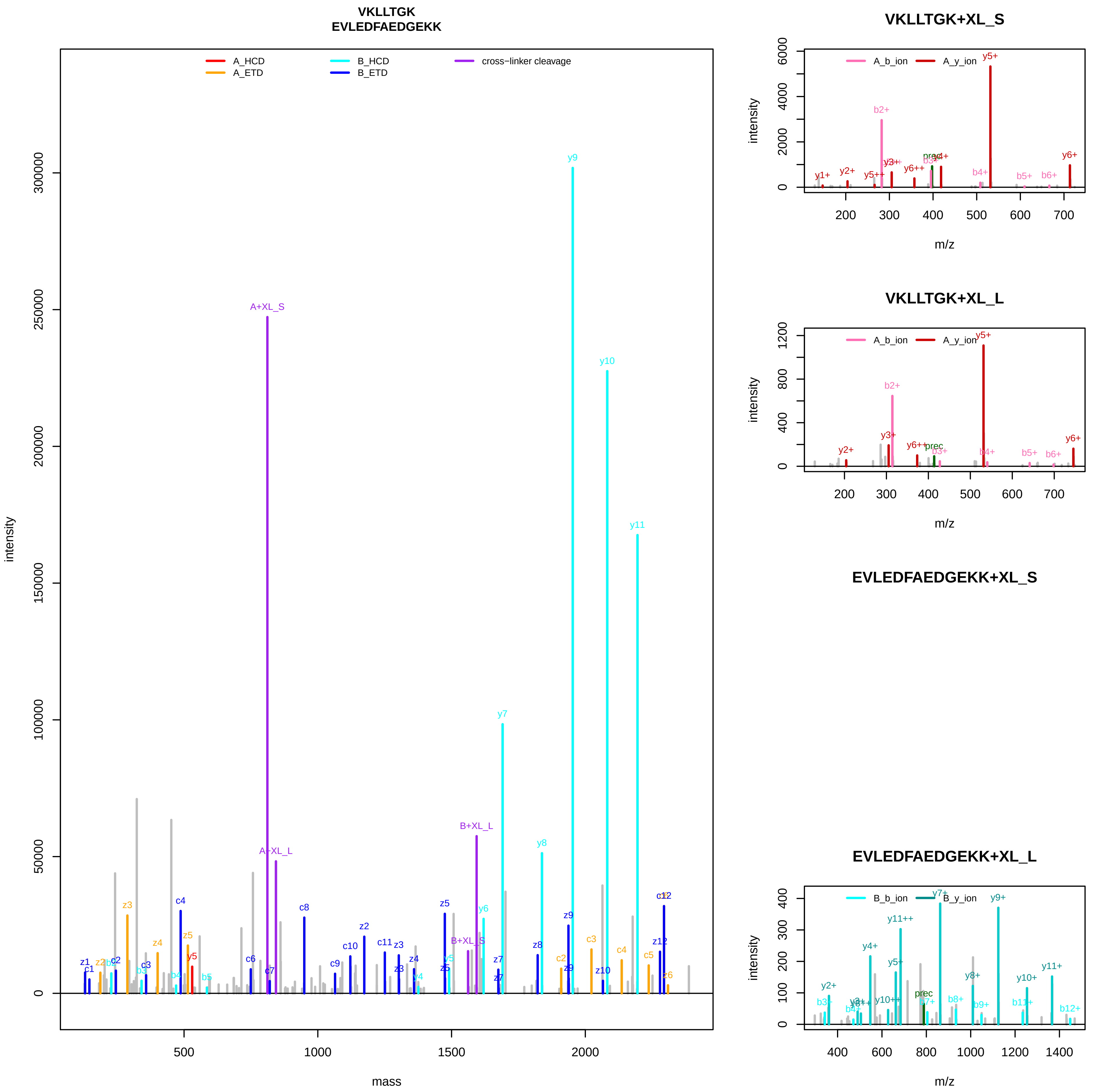
VKLLTGK
EVLEDFAEDGEKK
A_HCD
A_ETD
B_HCD
B_ETD
cross−linker cleavage
intensity
mass
0
50000
100000
150000
200000
250000
300000
500
1000
1500
2000
A+XL_S
A+XL_L
B+XL_L
B+XL_S
y9
y10
y11
y7
y8
y6
c12
z12
c8
c4
z5
z8
z9
z10
c2
c3
c4
c5
z6
VKLLTGK+XL_S
A_b_ion
A_y_ion
y5+
b2+
y6+
y1+
y2+
y5++
y3+
y6++
b3+
y4+
b4+
b5+
b6+
prec
m/z
VKLLTGK+XL_L
EVLEDFAEDGEKK+XL_S
EVLEDFAEDGEKK+XL_L
B_b_ion
B_y_ion
y7+
y9+
y11++
y4+
y5+
y2+
y8+
y10+
y11+
b3+
b4+
b7+
b8+
b9+
b11+
b12+
